# Microstructure and chemical composition of Roman orichalcum coins emitted after the monetary reform of *Augustus* (23 B.C.)

**DOI:** 10.1038/s41598-019-48941-4

**Published:** 2019-09-03

**Authors:** Melania Di Fazio, Anna Candida Felici, Fiorenzo Catalli, Caterina De Vito

**Affiliations:** 1 Department of Earth Sciences, Sapienza University of Rome, P. le Aldo Moro 5, Rome, Italy; 2 Department of Basic and Applied Sciences for Engineering, Sapienza University of Rome, Via Antonio Scarpa 16, Rome, Italy; 3 Via Attilio Friggeri 95, 00136 Rome, Italy

**Keywords:** Metals and alloys, Characterization and analytical techniques

## Abstract

A collection of ancient Roman orichalcum coins, *i*.*e*., a copper-zinc alloy, minted under the reigns from Caesar to Domitianus, have been characterised using scanning electron microscopy (SEM-EDS) and electron microprobe analysis (EMPA). We studied, for the first time, coins emitted by Romans after the reforms of *Augustus* (23 B.C.) and *Nero* (63–64 A.D). These coins, consisting of *asses*, *sestertii*, *dupondii* and *semisses*, were analysed using non- and invasive analyses, aiming to explore microstructure, corrosive process and to acquire quantitative chemical analysis. The results revealed that the coins are characterized by porous external layers, which are affected by dezincification and decuprification processes. As pictured by the X-ray maps, the elemental distribution of Cu and Zn shows patterns of depletion that in some cases penetrate in deep up to 1 mm. The composition of the un-corroded nucleus is a Cu-Zn alloy containing up to 30% of Zn, typical of coins produced *via* cementation process.

## Introduction

The term *Orichalcum* is a classical ancient word used to describe a Cu-Zn-based alloy with a discrete percentage of Zn (5–28%)^1,2^, and similar in appearance to the modern brass. Higher in value than the bronze, this alloy was melted in the form of ingots^3^ and then used to obtain different kind of objects both in the ancient^4–6^ and modern times^7^.

Orichalcum was rarely used for coinage in the Hellenistic world^8^ and was experimentally used by Romans under the reigns of *Caesar* and *Marcus Antonius*.

The scope of the monetary reform of *Augustus* (23 B.C.) was reducing the disorder of the Republican Roman coinage, introducing *sestertii* and *dupondii* in Cu-Zn alloy, rather than silver and bronze. Lately, a new monetary reform was made by *Nero* during the 63–64 A.D., introducing *asses*, *semisses* and *quadrantes* in orichalcum^9,10^.

The *cementation* process, used to produce the Cu-Zn alloy, was improved and developed by ancient Romans for the coinage and production of different kind of artefacts^1,2,11,12^. This process consisted of using crucibles with specific size and shape to reach thermodynamic requirements for orichalcum production. The diffusion of the vapours of zinc into the copper melt produced a Cu-Zn based alloy with a maximum content of Zn near 30%. The maximum content of zinc in orichalcum was mainly controlled by temperature, partial pressure of Zn_vapour_ and the redox conditions^12^. The transformation of zinc oxide into metal required (strongly) reducing conditions, with a minimum temperature of about 900 °C. This temperature threshold represents the boiling point of zinc, necessary to produce the vapour phase of zinc^1,13^. The re-oxidation process and the suitable partial pressure of zinc were provided during *cementation* process by the use of closed crucibles^13,14^.

Similar processes and techniques of that of Roman *cementation* were applied and diffused in Middle East^5^, India^15^, China^16,17^, Northern Europe^12,13,18^.

The corrosion of orichalcum coins as well as that of other archaeological artefacts is one of the most important problems linked to the conservation and the preservation of Cultural Heritage materials^5–7,19,20^.

The corrosion of orichalcum is associated with dezincification and decuprification processes^21–23^, which can occur for several microns in depth; therefore, to evaluate the degree of corrosion it is necessary to analyse cross section of samples “from rim-core-to-rim”^24,25^.

A very limited number of studies about orichalcum coins^6,19,21,23^ and their corrosion are reported and, above all, these contributions are based on the use of qualitative data or semi-quantitative data on dezincification process.

Nowadays, the most common techniques applied to investigate ancient alloys are Scanning Electron Microscope^6,26,27^ and X-Ray Fluorescence^22,28^.

A multi-analytical approach^29,30^ based on µ-Raman^31^, X-ray Diffraction, X-ray Photoelectron Spectroscopy^32^ or micro-PIXE^33^ was applied to investigate the external layers of metal object. Electrochemical analysis as Voltammetry of Immobilized Micro Particle and Electrochemical Impedance Spectroscopy^34–39^ are also used to explore the state of conservation of the patina and to identify the corrosion products.

Some authors applied superficial techniques to investigate both corroded and uncorroded alloy composing samples^40^. However, the error related to major and minor elements is too high to help answer to archaeological issues.

Nowadays, the most suitable way to investigate the depth of the dezincification process is to analyse cross sections obtained from samples in study (with the authorisation of the owners of the coins). Destructive analysis should be preferred, when possible, to evaluate the “real” composition of orichalcum, because the small amount of sample removed from the patina of the coins could not be representative of uncorroded alloy^2^. Cross-section analysis allows comparing the real quantity of major elements, which composes orichalcum produced in different age and, thus giving information about the development of the *cementation* process during the Roman Era.

Recently, EMPA technique was used obtaining quantitative chemical analysis of Roman alloys used for coinage^25^.

The aim of this study is to investigate the composition of the orichalcum coins in the un-corroded nucleus and the corrosion phenomena that affected these coins, acquiring qualitative and quantitative chemical data from the correded external layers to the unaltered core.

We use SEM and EMP analyses to reconstruct the nature of the original alloy and to explore the mechanisms which induced corrosion^6^. The patterns of corrosion are explored using SEM-EDS and X-ray imaging of the elements of the alloy. In addition, a deep investigation of the microstructure is made.

**Figure 1:**
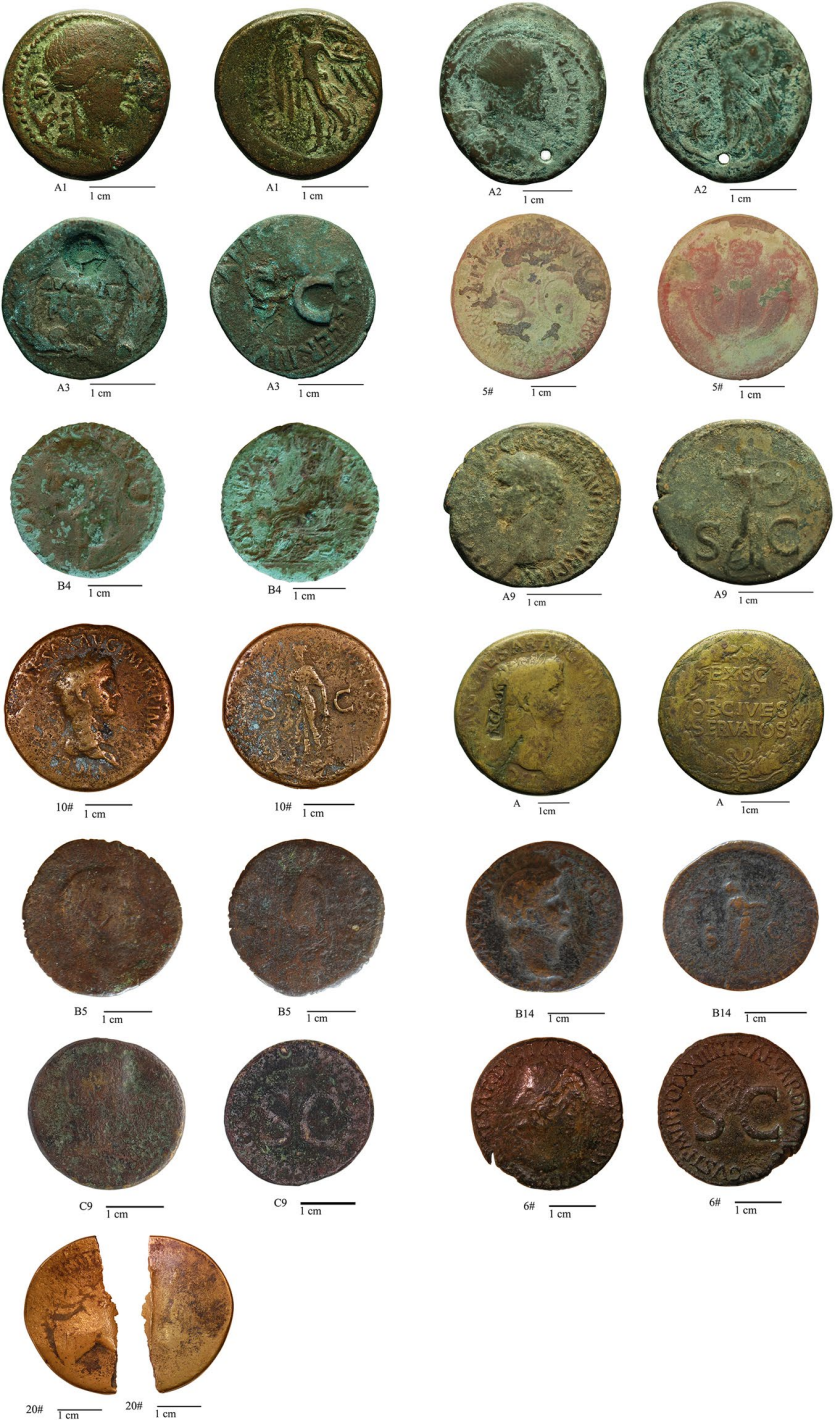
The orichalcum coins studied in this work. Numismatic information is reported in Table 1.

A set of 13 Roman orichalcum coins (Fig. 1 and Table 1) were selected for this study from about 60 coins from *Private Collections*. Numismatic examination, considering the weight, the size, the legend and the engraved type of each coin^41,42^, revealed that they are *asses*, *sestertii*, *dupondii* and *semisses* minted from *Julius Caesar* to *Domitianus*. The majority of coins were minted after the two important monetary reform of Augustus (23 B.C.) and Nero (63–64 A.D).

**Table 1:** Numismatic features of the coins.

Sample	Value (denomination)	Authorithy	Emission authority	year	Mint	Numismatic reference
A1	As	—	Q. Oppius	88 B.C.	Laodicea (Turkey)	RRC 550/1-2
A2	As	G. Iulius Caesar (100 B.C.-44 B.C.)	C. Clovius	45 B.C.	Uncertain	RRC 476/1-2
A3	Dupondius	Augustus (63 B.C. -14 A.D.)	C. Cassius Celer	16 B.C.	Rome	RIC I, 2 Augustus 375
5#	Sestertius	Tiberius (42 B.C.-37 A.D.)	—	22–23 A.D.	Rome	RIC I, 2 Tiberius 42
6#	Sestertius	Tiberius (42 B.C.-37 A.D.)	—	21–22	Rome	RIC I, 2 Tiberius 48 var.
B4	Dupondius	Caligula (12–41 A.D.)	—	37–41 A.D.	Rome	RIC I, 2 Gaius/Caligula 56
C9	As	Caligula (12–41 A.D.)	—	37–38 A.D.	Rome	RIC I, 2 Gaius/Caligula 35
A9	As	Claudius (10–54 A.D.)	—	41–50 A.D.	Rome	RIC I, 2 Claudius 100
10#	Sestertius	Claudius (10–54 A.D.)	—	41–50 A.D.	Rome	RIC I, 2 Claudius 99 (?)
A	Sestertius	Claudius (10–54 A.D.)		50–54 A.D.	Rome	RIC I, 2 Claudius 112
B5	Sestertius	Claudius (10–54 A.D.)	—	50–54 A.D.	Rome	RIC I, 2 Claudius 115
B14	Semis	Nero (37–68 A.D.)	—	62–68 A.D.	Rome	RIC I, 2 Nero 78
20#	Sestertius	Domitianus (51–96 A.D.)	—	—	—	—

Samples 6#, C9 and A9 are considered exotic samples.

Three samples turned out to be interesting cases of study: the two *asses* coined by *Caligula* and *Claudius* before the introduction of the *asses* in orichalcum with the *Nero* reform, and the *sestertius* coined by *Tiberius* with a Cu-based alloy, contrary to historical information that describes the *Tiberius sestertii* as a denomination in orichalcum. Indeed, one of the false certainties of the numismatists is the belief that orichalcum was used by *Mithridates VI* (120-96 B.C.) for a short time of his coinage^8^, lately experimental emissions by *Caesar* and *Marcus Antonio* occur. Finally, under the Julio-Claudian dynasty (from *Augustus* to *Nero*), a limited number of denominations (*sestertii*, *dupondii* and under *Nero* also *semisses* and *quadrantes*)^43^ were emitted in orichalcum.

This research attempts to fill the scientific gap existing in the Roman orichalcum coinage, contributing to characterize this ancient alloy, to better understand the dezincification process and to highlight possible differences among samples minted in different years.

## Results and Discussion

### XRF investigation for a preliminary discrimination of the coins

A preliminary XRF analysìs allows confirming that the majority of the samples are in orichalcum; although those affected by important dezincification process can be erroneously consider Cu-based alloys. This investigation is useful to test the presence of Zn in the surfaces even at low percentage, avoiding unnecessary cutting of the samples that are not in orichalcum. Indeed, deep corroded orichalcum coins are similar in appearance to that in bronze alloy.

Despite numismatic references, the screening by XRF on a non-orichalcum *sestertius*, minted under *Tiberius*, revealed the presence of zinc (sample 6#, RIC I, 2 48 var.). In addition, two *asses*, theoretically in bronze and minted during *Caligula* and *Claudius* Empires, seemed also to be composed of orichalcum (sample C9, RIC I, 2 Caligula 35, sample A9, RIC I, 2 48)^42^.

The surface analysis by XRF reveals the presence of Cu and Zn as two main alloy components, followed by Fe and Pb. Tin is present in all samples, with higher peaks particularly in A2, A and B14 samples than others. Chlorine occurs in all samples (Fig. 1S), but in low amounts as highlighted by the low-intensity of its characteristic peaks. Exogenous elements, such as Ca, S, Si, Al, P and Mn are also present^19^ and represent contaminants from soils through porosity of the external layer.

### SEM investigation of the microstructure, qualitative chemical composition and elemental distribution

**Figure 2:**
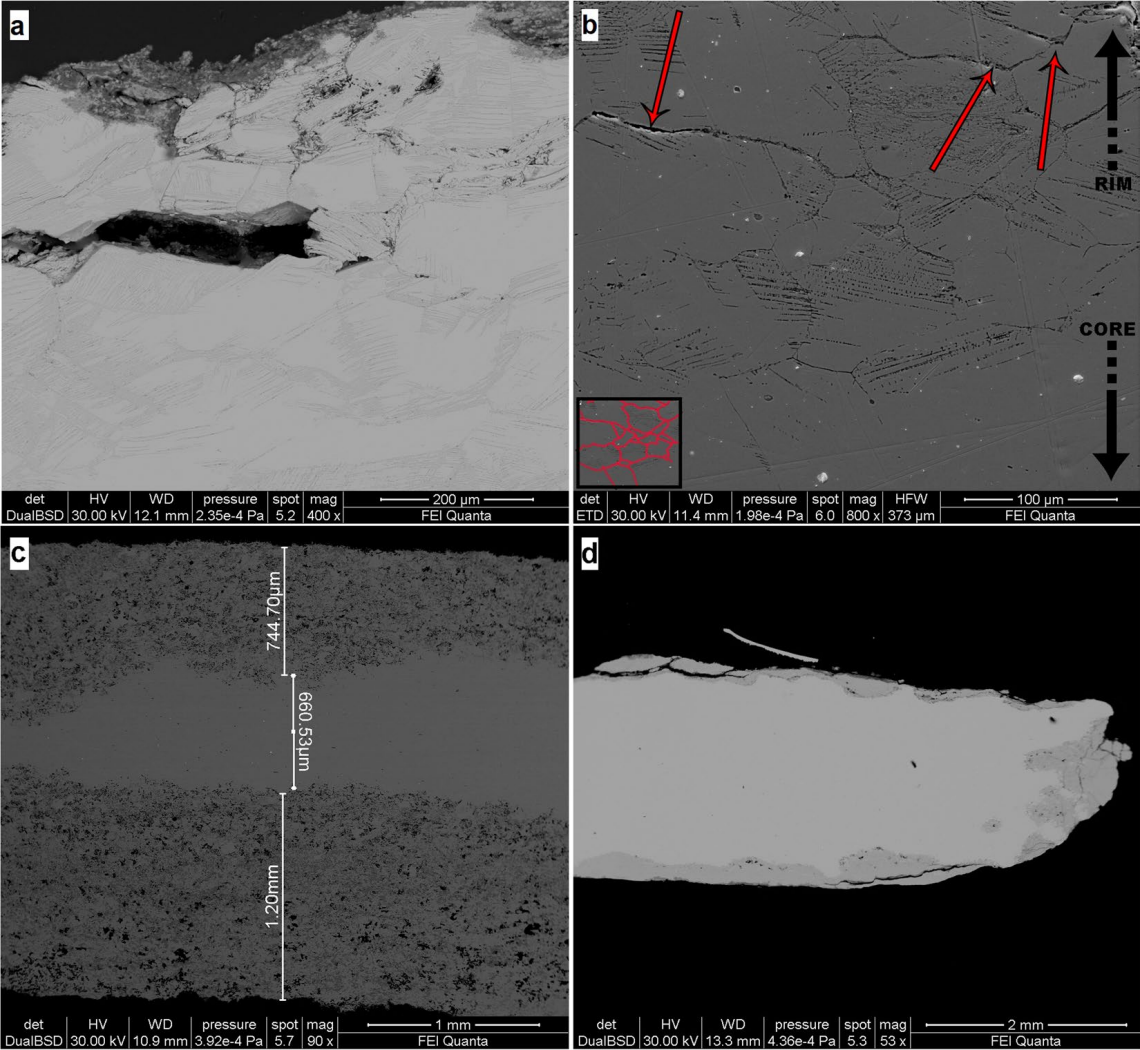
BSE images of coins B4(a), 5#(c) and B14(d) and SE image of coin A3(b). The α-grains structure and the strain lines are evident in (**a**) and (**b**). The process of dezincification is showed in (**c**) and (**d**), reaching 1.2 mm as the maximum depth of corrosion (**c**). The red arrows in (**b**) indicate the trans-granular stress corrosion.

The results on cross-sections analyses of all the coins allow studying microstructure and compositional variations along the thickness of the coins. The microstructure of the alloy presents (SE image at high magnification) the typical α grains structure, clearly visible near the external rim of the sample A3 (Fig. 2b). This is the result of an efficient casting-cooling practice, suggesting a well-controlled *cementation* process^44^. The grains size varies from ~50 µm to ~300 µm, suggesting a non-rapid cooling speed. Moreover, some grains present deformed borders with thin strain lines inside (Fig. 2a, sample B4 and Fig. 2b, sample A3), due to heavy cold-working, which causes of the slip of crystal planes with the result of a series of parallel movements that produced fine lines^44,45^. In addition, the twin lines do not present further deformation, suggesting that coins were struck only once. The dendritic structures are not observed.

We present and discuss here the results of samples 5# and B14 (Fig. 2c,d) from the external corroded layers to the inner core, having high and low degree of corrosion, respectively. In addition, the chemical composition of the unaltered area gives information about the ratio of the two main metals in the alloy.

**Figure 3:**
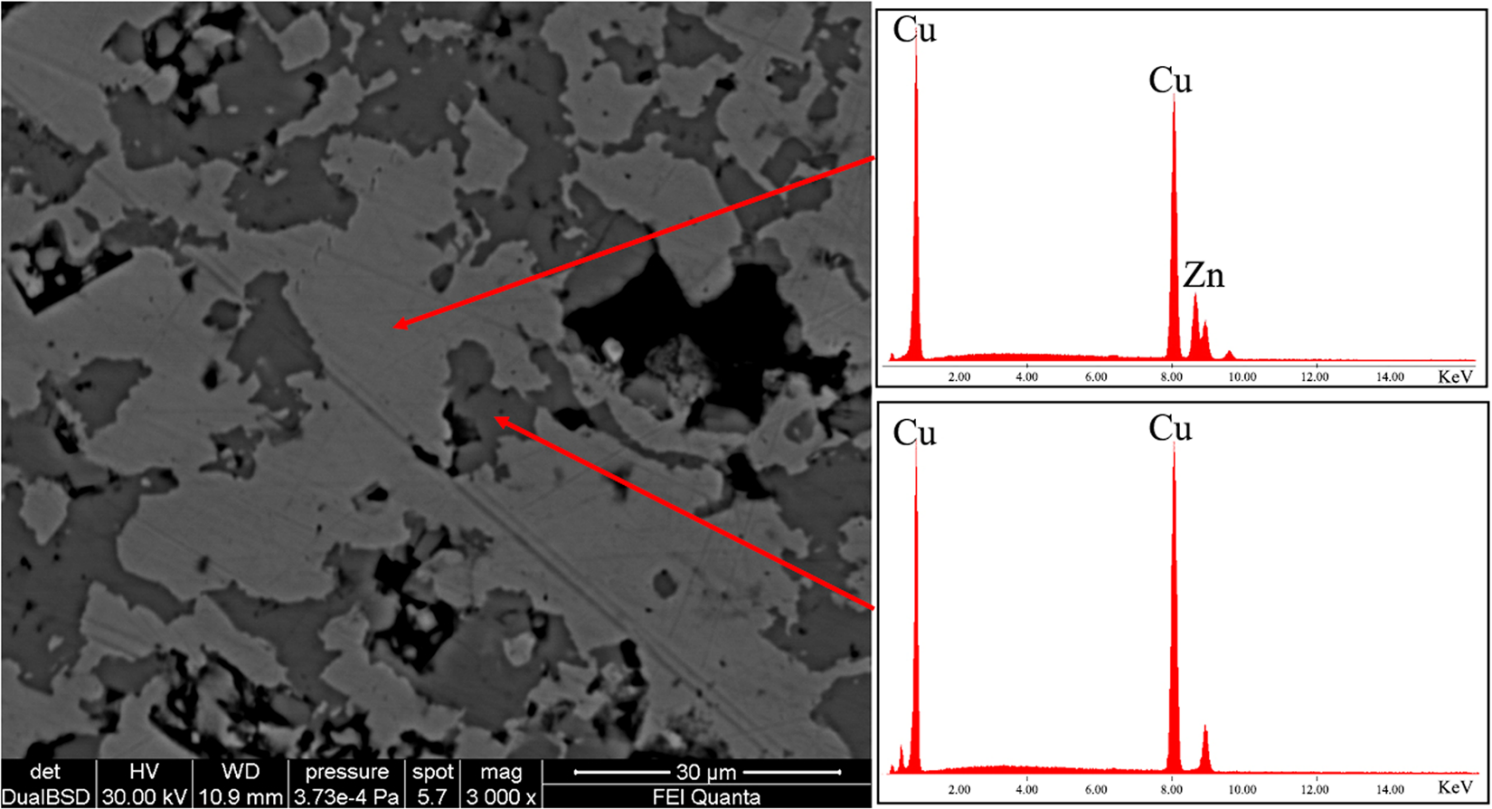
BSE image of the external patina of sample 5# in cross section. ESD spectrum acquired on the lighter area shows a Cu-Zn alloy and that of the darker areas shows only Cu, due to the loss of Zn.

The corroded patina has an irregular thickness with different degree of dezincification, followed by high porosity in the external areas. Sample B14 (Fig. 2d) has two different layers of patina, *i*.*e*., the external one (darker grey) has a thickness of few microns with partial detachments from the sample; the second layer (medium grey) is more coherent with the un-corroded core (lighter grey). On the contrary, the patina of the sample 5# (Fig. 2c) has one layer with an irregular surface in shape. In this sample an important dezincification process occurs, extending up to 1.2 mm in depth. The corrosion proceeds from the rim to the inner core with micro-areas of selective corrosion (Fig. 3, BSE image), driven by grain boundaries of the α-grain structure^6^ of the alloy. This selective corrosion is evident in sample 5# (Fig. 3), where the content of Zn in corroded micro-domains (darker grey) is below the 20%; whereas in the lighter grey micro-domains Zn content is similar to that of un-corroded nucleus (Table 2). The selective dealloying can be attribute to the trans-granular stress corrosion, usually observed in alloys with content of Zn between 20% and 30%^46^ and can be clearly observed at the contact of the border zones of the sample A3 (Fig. 2b, red arrows). With the evolution of the dezincification process in the external layer is formed a sponge-type structure, typical of brass with low content of Sn, as observed by Constantinides *et al*.^47^ in standard sample representative of archaeological brass. Moreover, in the most corroded areas of this layer porous microstructure was found as reported also in previous studies^21,48^ mainly made by copper oxides.

**Table 2:** Quantitative EMP analyses of major element of the sample 5#. Data of darker and lighter grey areas are referred to BSE image of Fig. 4.

Element (w.t. %)	Upper corroded layer		Un-corroded core		Lower corroded layer	
	Darker grey	Lighter grey	Upper point	Lower point	Darker grey	Lighter grey
Cu	91.78	86.41	77.17	77.28	99.05	93.63
Zn	6.98	12.42	23.49	23.38	0.60	5.42

In addition, qualitative EDS analysis (Fig. 3, EDS spectra) were carried out to evaluate the chemical composition of the unaltered core of the coins. EDS spectra confirmed that Cu and Zn are the two major elements composing the alloy. Lead is present in samples A1, A2, B5 and 20#, whereas Sn in samples A3, 20#, occurring these elements as minor and/or trace. Iron occurs in A1, A2, A, B4, 5#, 20#.

**Figure 4:**
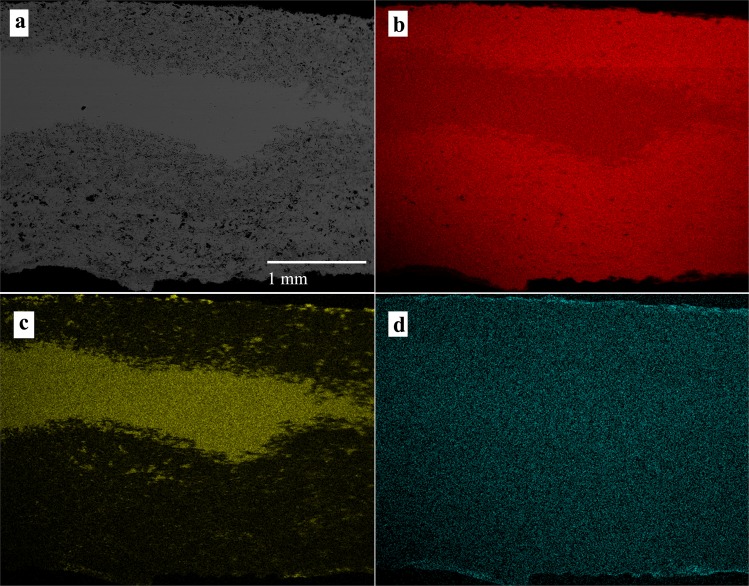
BSE image of sample 5# (**a**) and X-ray maps of Cu (**b**), Zn (**c**) and Fe (**d**).

**Figure 5:**
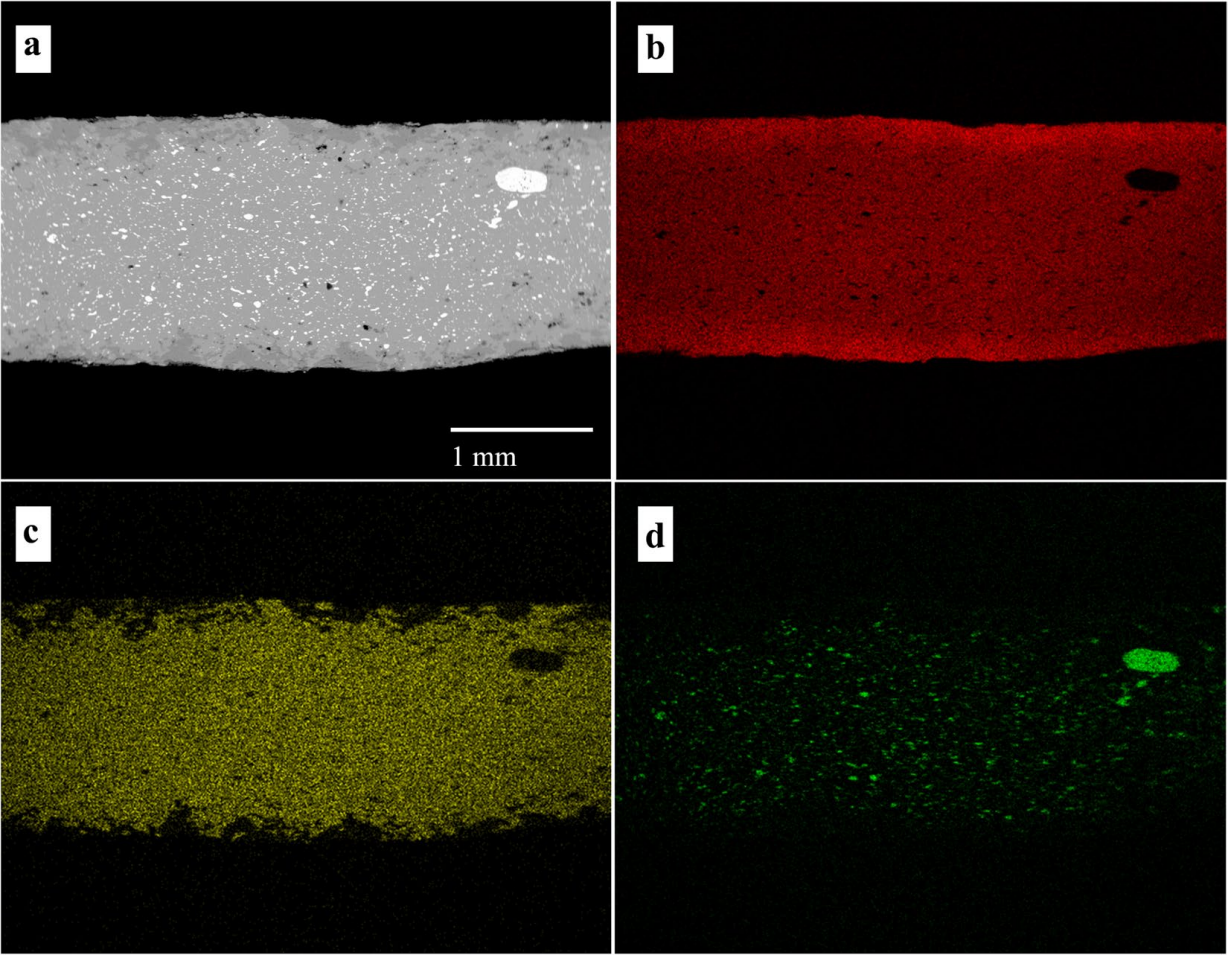
BSE image of sample B5 (**a**) and X-ray maps of Cu (**b**), Zn (**c**) and Pb (**d**).

X-ray maps of the elements composing the alloy provide information about their distribution along rim-core-rim cross-sections. The maps of samples 5# and B5 (Figs 4 and 5) illustrate two different degrees of dezincification, revealed studying the whole coin set: a high level of corrosion, for example in sample #5 (Fig. 4) and a medium level of corrosion, as in sample B5 (Fig. 5). The core of almost all the samples, as shown in the X-ray maps (Fig. 4 and Fig. 5), presents a homogeneous distribution of Cu, Zn and, where present, also of Sn. These patterns indicate an expertise in *cementation* process by Romans and a well-controlled procedure during the melting-cooling phase of the alloys in the Roman mint, with a diffusion of Zn at nanoscale level along with the absence of micro-domains of Cu and Zn. Lead, when present in the alloy, forms droplets of different size throughout the coin, due to the low solubility of these two metallic elements, which at low temperature do not give solid solution^18^. The homogeneous presence of Fe (Fig. 4) suggests the use of chalcopyrite rich-ore for the extraction of Cu. However, the superficial Fe-enrichment can be explained with the contribution of exogenous Fe from the soils. The occurrence of Pb suggests the use of raw materials composed of a mix of base-metals, such as chalcopyrite, galena and sphalerite or the use of calamine from carbonate-hosted Zn (Pb) ore deposits^49^. As previously revealed by XRF analyses, the occurrence of exogenous Cl probably induced corrosion processes, producing pitting on the surface of the coins, due to the high pitting potential of the Cu-Zn alloy attributed to the presence of Cu_2_O and ZnO on the coins’ patina^50^.

The rim of the coins, as showed in the X-ray maps (Figs 4 and 5), presents evidence of dezincification process, involving Cu enrichment in the external surfaces.

### SEM investigation of the exotic samples

The three exotic samples, *asses* C9, *asses* A9 and *sestertius* 6#, were also investigated using SEM technique.

**Figure 6:**
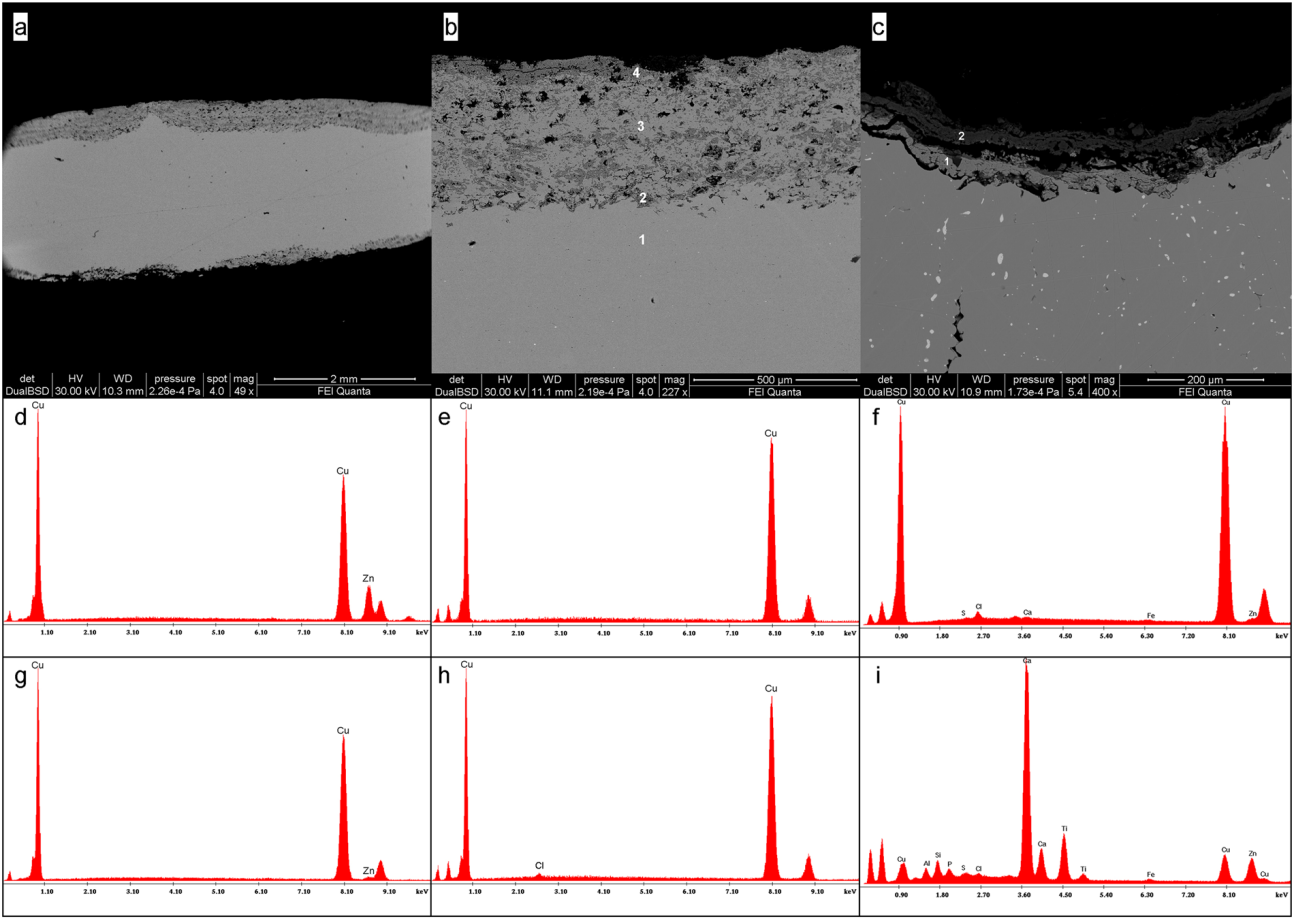
BSE images of exotic samples C9 (**a**,**b**) and A9 (**c**). The EDS spectra of sample C9 in points 1, 2, 3 and 4 (**d**–**h**) were acquired in spot analyses as highlighted in (**b**). The EDS spectra of sample A9 were acquired in points 1 and 2 (**f**,**i**) as highlighted in (**c**).

As other orichalcum samples, C9 presents a corroded patina extended up to ~400 µm in depth, with a medium degree of porosity (Fig. 6a). Also in this case, the corroded area is characterized by micro-domains with different amount of Zn, due to a selective de-alloying. However, the distribution of the corroded micro-domains follows a banded structure (Fig. 6a,b) in which high dezinced areas (darker grey) are alternated to low dezincificated areas (lighter grey). Qualitative EDS analysis confirms the presence of the corroded bands if compared to the one of the uncorroded area. Indeed, the unaltered alloy presents the typical Cu-Zn composition of the orichalcum (Fig. 6d, EDS spectrum corresponding to point 1 in Fig. 6b), whereas the corroded bands are Zn depleted (Fig. 6e,h EDS spectra corresponding respectively to points 2 and 4 in Fig. 6b) and the bands with a low degree of dezincification (Fig. 6b, point 3) present an intermediate composition between the unaltered alloy and the corroded areas (Fig. 6g EDS spectrum).

Sample A9 presents a remarkable detachment of the patina from the sample (Fig. 6c). The patina is composed of two different layers, where the external one presents a higher degree of corrosion than the inner layer. EDS analysis shows the presence of exogenous elements, such as C, O, Al, Si, Ca, P, S, Ti and Fe as contaminants from soils, on the external surface and inside the empty space derived from the detachment of the patina (Fig. 6i, EDS spectrum corresponding to point 2 in Fig. 6c). The presence of Zn showed by EDS spectrum of the inner layer (Fig. 6f, EDS spectrum corresponding to point 1 in Fig. 6c) suggests a lower degree of corrosion, and a less presence of contaminants elements, probably due to the protective effect induced by the outer passive layer.

Also Cl occurs in both the corroded layers of the patina (Fig. 6f,i), inducing the corrosion process and in turn the detachment of the layers from the sample.

Sample 6# is one of the two *sestertii* minted during the reign of *Tiberius* here studied (the other one is sample 5#). The alloy of this sample is a Cu-Sn-based alloy (bronze), contrary to the rules of the monetary reform of Augustus (23 B.C.), and presents a homogeneous pattern through the section (rim-core-rim analysis with SEM-EDS). The Cu depleted patina is extended up to ~120 µm in depth and results particularly corroded in the intergranular zone. Chlorine is present in the areas where Cu depletion occurs, and an high porosity characterizes the patina.

### EMP analysis of the un-corroded nucleus

EMP analysis of the un-corroded nucleus allows obtaining quantitative chemical compositions of the coins and along with SEM imaging and X-ray maps permits to reconstruct the dezincification and decuprification pattern through the section (rim-core-to-rim). This analysis was necessary to determinate the composition of the orichalcum alloy without the influence of the corroded external surfaces and to highlight differences among alloys casted under different Emperors.

**Figure 7:**
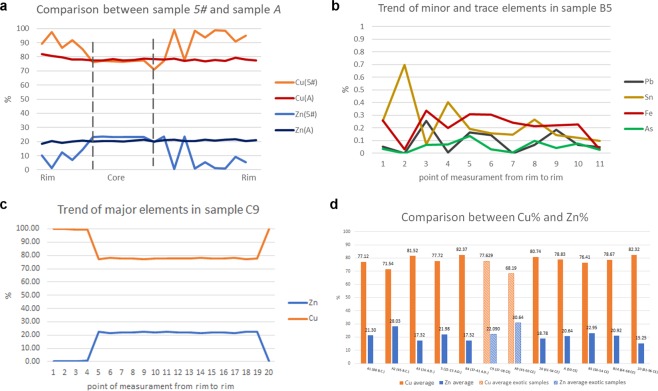
Patterns of EMP analysis on cross section and chronological comparison among samples. (**a**) The line-graph represent the comparison between the pattern of Cu and Zn in samples 5# and A. Dashed lines describe the area of the uncorroded core of sample 5#; (**b**) line-graph representing the trend of minor and trace elements (Pb, Sn, Fe and As) in sample B5; (**c**) line-graph representing the trend of Cu and Zn in sample C9; (**d**) bar-plot of chronological comparison of the average of major elements in orichalcum coins. The bars with a line pattern represent the average Cu or Zn values of the exotic samples. Data were obtained considering the uncorroded core of each sample, using a minimum of 8 to 22 spot analyses.

Samples with low degree of corrosion show homogeneous distribution of Cu% and Zn% throughout the section (*i*.*e*., sample A), whereas in the corroded samples the percentage of Zn varies from 1.33% to 23.49% (sample 5#, Fig. 7a). The exotic coin C9 presents trend of values of the two main elements, from one rim to the other, comparable to samples with high degree of corrosion (Fig. 7c). Indeed, the first 4 point of measurements (400 µm ca.) have 98–99% of Cu and values of Zn < 0.5%. This confirms the high degree of dezincification of the sample. In the uncorroded core of the coin, the alloy is composed mainly of 77% ca. of Cu and 20% ca. of Zn, whereas in the last 100 µm of the cross section the values of the major elements return to having the values of the other side patina. The exotic coin A9 does not presents compositional variation of Cu and Zn in the whole section, confirming the low degree of corrosion of such sample. Moreover, A9 is composed of the alloy with the maximum average value of Zn (30.64%) of the whole collection.

Minor and trace elements such as Sn, As and Fe, present homogenous values in un-corroded areas of the alloy (Fig. 7b), whereas Pb is often heterogeneous as in some samples it occurs as exsolution domains from ~400 µm to ~5 µm resulting in Pb-rich areas (Fig. 5). The low content of Fe and their trend from rim to rim inside the samples can suggest the use of chalcopyrite to extract Cu (Table 3).

**Table 3:** Maximum (Max.), minimum (Min.) and average (Avg.) EMP analyses of major, minor and trace elements.

Element (w.t.%)	A1			A2			A3			5#			B4			C9		
	Max.	Min.	Avg. n = 13	Max.	Min.	Avg. n = 15	Max.	Min.	Avg. n = 10	Max.	Min.	Avg. n = 8	Max.	Min.	Avg. n = 8	Max.	Min.	Avg. n = 15
Cu	79.42	72.52	77.12	72.01	71.27	71.54	82.12	81.14	81.52	85.30	76.21	77.72	82.87	82.05	82.37	78.30	77.12	77.63
Zn	22.34	19.75	21.30	28.28	27.59	28.03	17.82	16.73	17.32	23.49	14.46	21.98	17.65	16.89	17.32	22.56	21.40	22.09
Pb	5.49	0.00	0.45	0.20	0.00	0.06	0.09	0.00	0.03	0.09	0.00	0.03	0.11	0.00	0.04	0.07	0.00	0.02
Fe	0.47	0.21	0.32	0.29	0.19	0.24	0.40	0.25	0.34	0.17	0.03	0.13	0.11	0.03	0.07	0.21	0.12	0.18
Sn	1.14	0.35	0.73	0.09	0.00	0.06	0.84	0.61	0.72	0.13	0.04	0.09	0.18	0.09	0.02	0.11	0.00	0.02
As	0.09	0.00	0.03	0.09	0.00	0.04	0.04	0.00	0.01	0.06	0.00	0.02	0.10	0.00	0.05	0.06	0.00	0.02
**Element (w.t.%)**	**A9**			**10#**			**A**			**B5**			**B14**			**20#**		
	**Max**.	**Min**.	**Avg. n** = **18**	**Max**.	**Min**.	**Avg. n** = **14**	**Max**.	**Min**.	**Avg. n** = **22**	**Max**.	**Min**.	**Avg. n** = **8**	**Max**.	**Min**.	**Avg. n** = **9**	**Max**.	**Min**.	**Avg. n** = **11**
Cu	69.75	67.61	68.19	81.19	80.22	80.74	81.01	77.72	78.83	76.98	75.55	76.41	79.00	78.44	78.67	83.47	81.99	82.32
Zn	31.25	29.29	30.64	19.03	18.45	18.78	21.68	18.38	20.64	23.73	22.40	22.95	21.41	20.65	20.92	15.02	15.41	15.25
Pb	0.10	0.00	0.02	0.77	0.00	0.12	0.17	0.00	0.06	0.25	0.00	0.11	0.10	0.00	0.03	0.06	0.00	0.03
Fe	0.05	0.00	0.01	0.24	0.17	0.21	0.38	0.27	0.34	0.34	0.20	0.25	0.31	0.20	0.24	0.32	0.34	0.28
Sn	1.36	0.68	1.08	0.13	0.02	0.06	0.15	0.01	0.07	0.40	0.07	0.19	0.07	0.00	0.04	2.27	1.00	2.03
As	0.10	0.00	0.01	0.10	0.02	0.06	0.05	0.00	0.01	0.13	0.01	0.06	0.15	0.00	0.07	0.05	0.00	0.01

EMPA data acquired in the uncorroded core of each coin are compared with the average values of Caley^51^, from this comparison our data show an irregular fluctuation of Cu and Zn through 100 years of minting of orichalcum coins (Fig. 7d). Therefore, the fluctuations of Cu and Zn seem to suggest difficulties in the supply of raw materials as well as not regular control of the *cementation* process to produce orichalcum ingots.

Previous studies^51^ suggested the possibility to approximately date coins, and other orichalcum objects, comparing the content of Zn in the alloy with that of a series of dated coins. Caley described a decrease of the Zn that corresponded to a regular increase in the average proportion of Sn and Pb, from the oldest to the most recent coins (23 B.C. - 162 A.D.). He justified the chronological decrease of the Zn with the necessity of recasting of metals. However, comparing the private collection analysed in the present study with the results of Caley^51^, the chronology by Cu and Zn percentages results different. Indeed, the average value of Zn, from 88 B.C. to 96 A.D., results extremely variable and fluctuating (Fig. 7d), exceeding sometimes the value of 28% considered as the maximum limit possible in ancient brasses by some authors^44^.

## Methods

All the information on samples, concerning year and place of coinage, emission authority and numismatic references, is reported in Table 1.

Preliminarily, qualitative elemental analyses on the patinas were carried out by energy-dispersive X-ray fluorescence spectroscopy (EDXRF) (Department of Basic and Applied Sciences for Engineering, Sapienza University of Rome, Italy). The spectrometer consists of an X-ray generator (Amptek MiniX) with an anode target of rhodium and a beryllium window of 127 µm of thickness. The detector is a Peltier cooled silicon drift with integrated amplifier and multi-channel analyzer (Amptek 123-SDD). The detector has a surface of 25 mm^2^, a thickness of 450 µm, a beryllium window of 12.5 µm of thickness and its energy resolution is 140 eV, full width half maximum at 5.9 keV. The incident and the revealed beams form an angle of 45° with respect to the surface of the sample. The analysed surface is distant 3 cm from the X-ray generator anode and 3.5 cm from the detector surface. The X-ray generator was equipped with a 2 mm diameter collimator and was powered with an accelerating potential difference of 35 kV and an electronic current of 15 µA. For each sample, three measures were obtained, with an acquisition time of 200 s each.

SEM investigation on cross section from rim to core of samples was performed using a FEI-Quanta 400 (SEM-EDS) instrument, operating at 30 kV, equipped with X-ray energy-dispersive spectroscopy (Department of Earth Sciences, Sapienza University of Rome, Italy). X-ray maps and SE/BSE imaging were carried out to investigate microstructure of the alloy, to estimate the depth of the corrosion and the elemental distribution from the external rim to core.

EMPA data for quantitative chemical analyses were performed using a Cameca SX50 electron microprobe equipped with five wavelength-dispersive spectrometers (CNR–IGAG, Rome, c/o Department of Earth Sciences, Sapienza University of Rome). The operating conditions were: accelerating voltage 15 kV, beam current 15 nA. Element peaks and background were measured with counting times of 20 and 10 s respectively. Metallic Cu and metallic Zn were used respectively as a reference standard for Cu and Zn (LIF), galena for Pb (PET), cassiterite for Sn (PET), metallic Ni and metallic Co respectively for Ni and Co (LIF), synthetic GaAs for As (TAP), rhodonite and metallic Mn for Mn (PET), olivine and synthetic magnetite for Fe (LIF). Matrix corrections were calculated by the PAP method^52^, with software supplied by Microbeams Services. The detection limits under the specified working condition vary from 0.05 to 0.1 wt% with standard deviations from 0.02 to 0.04 wt%. The analytical error was ∼1% rel. for the major elements.

## Supplementary information

Figure 1s
