# Supplementary material for: Microstructure and chemical composition of Roman orichalcum coins emitted after the monetary reform of Augustus (23 B.C.)
Source: Sci Rep. 2019 Sep 3;9:12668. doi: 10.1038/s41598-019-48941-4 (PMC6722059; doi:10.1038/s41598-019-48941-4)
Supplement: Supplementary file 1 — Figure 1s [file 41598_2019_48941_MOESM1_ESM.pdf]

Microstructure and chemical composition of Roman orichalcum coins emitted after the monetary reform of *Augustus* (23 B.C.)

Melania Di Fazio[a], Anna Candida Felici[b], Fiorenzo Catalli[c] and Caterina De Vito[a*]

[a]Department of Earth Sciences, Sapienza University of Rome, P. le Aldo Moro 5, Rome, Italy
[b]Department of Basic and Applied Sciences for Engineering, Sapienza University of Rome, Italy
[c]Via Attilio Friggeri 95, 00136, Rome, Italy.

Corrisponding author: caterina.devito@uniroma1.it

Supplementary information

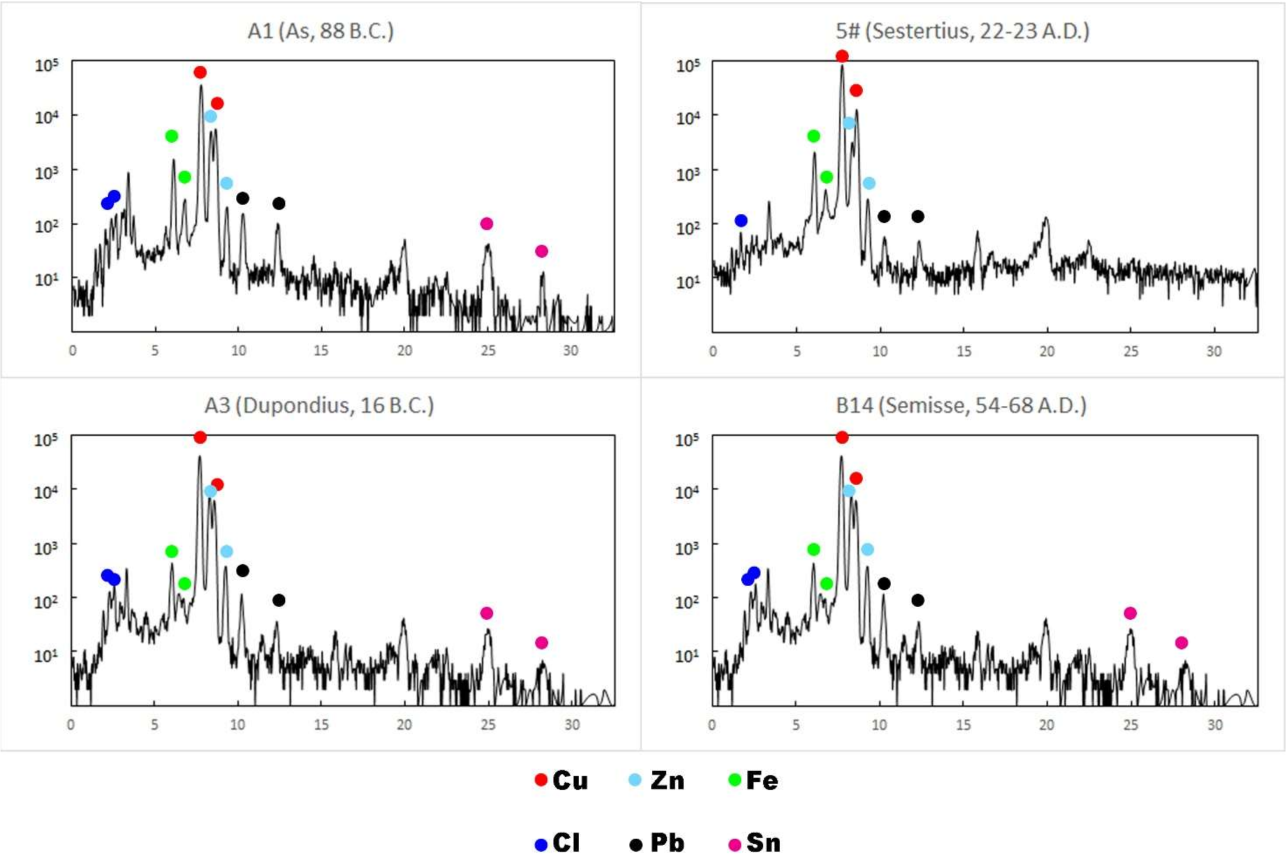

Figure 1S: XRF spectra of samples A1, A3, 5# and B14.
